# Alkaloids as Potential Phytochemicals against SARS-CoV-2: Approaches to the Associated Pivotal Mechanisms

**DOI:** 10.1155/2021/6632623

**Published:** 2021-05-13

**Authors:** Mohammad Bagher Majnooni, Sajad Fakhri, Gholamreza Bahrami, Maryam Naseri, Mohammad Hosein Farzaei, Javier Echeverría

**Affiliations:** ^1^Student Research Committee, Kermanshah University of Medical Sciences, Kermanshah, Iran; ^2^Pharmaceutical Sciences Research Center, Health Institute, Kermanshah University of Medical Sciences, Kermanshah, Iran; ^3^Medical Biology Research Center, Health Technology Institute, Kermanshah University of Medical Sciences, Kermanshah, Iran; ^4^Departamento de Ciencias del Ambiente, Facultad de Química y Biología, Universidad de Santiago de Chile, Santiago, Chile

## Abstract

Since its inception, the coronavirus disease 2019 (COVID-19) pandemic has infected millions of people around the world. Therefore, it is necessary to find effective treatments against Severe Acute Respiratory Syndrome Coronavirus-2 (SARS-CoV-2), as it is the viral source of COVID-19. Alkaloids are one of the most widespread plant-derived natural compounds with prominent antiviral effects. Accordingly, these phytochemicals have been promising candidates towards discovering effective treatments for COVID-19. Alkaloids have shown potential anti-SARS-CoV activities via inhibiting pathogenesis-associated targets of the Coronaviridae family that are required for the virus life cycle. In the current study, the chemistry, plant sources, and antiviral effects of alkaloids, as well as their anti-SARS-CoV-2 effect with related mechanisms, are reviewed towards discovering an effective treatment against COVID-19.

## 1. Introduction

Since the onset of coronavirus disease 2019 (COVID-19), extensive efforts have been made to find an effective drug/vaccine against the causative virus Severe Acute Respiratory Syndrome Coronavirus-2 (SARS-CoV-2) [1]. Considering the potential of medicinal plants and related isolated phytochemicals in targeting various diseases, including viral diseases, they could be helpful candidates in the treatment of COVID-19 [2–4]. Several studies are investigating the effectiveness of medicinal plants and their phytochemicals against SARS-CoV [5]. By containing one or more nitrogen atom(s) in their structure, alkaloids are one of the most widespread phytochemicals in plant families such as Amaryllidaceae, Apocynaceae, Papaveraceae, Asteraceae, and Solanaceae possessing potential biological activities and pharmacological effects [6, 7]. Besides, several studies have shown the prominent effects of alkaloids on various types of viruses such as influenza viruses, herpes simplex virus, human immunodeficiency virus, and hepatitis C virus [8, 9]. Also, previous *in vitro* and *in silico* studies have indicated the prominent effects of alkaloids against coronaviruses, especially SARS-CoV-2. The virus employs different mechanisms such as inhibition of the main protease (Mpro) and RNA-dependent RNA polymerase (RdRp), as well as interaction with coronavirus-associated structural proteins [10, 11]. This study aims to review the antiviral effects of alkaloids, especially against SARS-CoV-2, as well as their mechanisms regarding discovering effective compounds for the treatment of COVID-19.

## 2. Chemistry and Natural Sources of Alkaloids

Alkaloids are a wide group of naturally occurring organic compounds being a major part of the largest group of plant secondary metabolites [12]. The main characteristic feature of alkaloids is the presence of nitrogen atom/atoms in a negative oxidation state at their structures [13], which causes their alkaline properties with associated therapeutic effects [12–14]. From the synthesis point of view, most alkaloids are derived from a restricted number of amino acid precursors such as phenylalanine, tyrosine, tryptophan, ornithine, and lysine. These precursors are converted to versatile central intermediates, which results in the production of various alkaloids. When plant species contain more than 0.001% of alkaloids, they are considered as alkaloids sources. Accordingly, Amaryllidaceae, Apocynaceae, Papaveraceae, Asteraceae, Solanaceae, Rutaceae, Fabaceae, and Rubiaceae are some plant families with the potential of being used for pharmaceutical purposes [15].

Many alkaloids are used in human diet, both in food and drinks, such as alkaloids available in coffee seeds (caffeine), cacao seeds (theobromine and caffeine), tea leaves (theophylline and caffeine), tomatoes (tomatine), and potatoes (solanine) [8]. In addition to their edible uses, alkaloids also play a prominent role in human medical history and are widely used for the treatment of various diseases such as neurological disorders [16], cancer [17], metabolic disorder [18], and infectious diseases [19].

## 3. Antiviral Activities of Alkaloids

Plant-derived alkaloids have been widely considered as potential antiviral sources. In this regard, the effectiveness of alkaloids from *Corydalis saxicola* Bunting (Papaveraceae) was evaluated against hepatitis B virus. Eight protoberberine-type alkaloids were isolated from the methanolic (MeOH) extracts of this herb. These alkaloids showed inhibitory effects on the activity of HBsAg (hepatitis B surface antigen) and HBeAg (hepatitis B antigen) [20].

O'Rourke and coworkers investigated the inhibitory effects of alkaloids extracted from the sponge *Stylissa carteri* (Scopalinidae) on Human Immunodeficiency Virus (HIV-1). They detected three metabolites termed debromohymenialdisine (DBH), hymenialdisine (HD), and oroidin with anti-HIV-1 activities. Nuclear factor-kappa B (NF-*κ*B), CDK2, and G2-checkpoint interference were shown as three proposed therapeutic targets towards explaining the inhibitory effects of HD and DBH. Besides, while oroidin inhibited the retroviral reverse transcriptase, DBH and HD were ineffective [21]. *Lycoris radiata* (L'Hér.) of Amaryllidaceae is known for its antiviral potential against influenza virus type A. Four alkaloids, lycorine, hippeastrine, hemanthamine, and 11-hydroxyvittatine, showed antiviral activities against avian influenza virus H5N1 after virus entry into cells. Lycorine and hemanthamine also exhibited more potent antiviral activities, due to their inhibitory effects on nuclear-to-cytoplasmic export of the viral ribonucleoprotein complex, which play important roles in viral generation. Thus, Amaryllidaceae alkaloids seem to be potential anti-influenza agents [22].

Traditional Chinese herbal medicine is another source of alkaloids with antiviral activities. Nakamura et al. investigated the antiviral activities of 30 Chinese medicinal plants against herpes simplex virus- (HSV-) 1. They found that *Stephania cepharantha* Hayata (Menispermaceae) was the most potent antiviral plant and then isolated 49 alkaloids from its MeOH extract. Among those extracted ones, seventeen alkaloids were of thirteen bisbenzylisoquinolines, two morphinanes, one proaporphine, and one protoberberine possessing anti-HSV activities [23]. In another study, the effects of novel synthesis derivatives of quinolizidine alkaloid (-)-cytisine were studied against human influenza virus A (H1N1) and parainfluenza virus type 3 (HPIV3). As the result, 9-carboxamides of methylcytisine compounds were the most effective anti-H1N1 agents, which was in line with the result of *in silico* interactions with the Protein Data Bank code 4R7B active site of influenza A virus neuraminidase. Furthermore, derivatives with allyl isocyanate and (-)-cytisine or 9,11-dibromocytisin exerted inhibitory effects in HPIV3 reproduction [24].

The methanolic extract of the root bark of *Schumanniophyton magnificum* (K.Schum.) Harms (Rubiaceae) was tested to evaluate its antiviral activities against HIV and HSV, resulted in finding schumannificine 1 and other chromone alkaloids and acyl and methyl derivatives. Among those alkaloids, schumannificine 1 exhibited the highest anti-HIV activity due to its structural existence of piperidine ring and unsubstituted hydroxy groups, while a number of its derivatives exhibited anti-HSV activities [25]. In another study, two new and six known carbazole alkaloids from *Clausena anisum-olens* (Blanco) Merr. (Rutaceae) were evaluated on the HIV. They reported the highest antiviral activity against HIV virus for one of the new carbazole alkaloids [26]. Four pairs of enantiomers named (±)-*β*-myrifabral A, (±)-*α*-myrifabral A, (±)-*β*-myrifabral B, and (±)-*α*-myrifabral B were derived from *Myrioneuron faberi* Hemsl. ex F.B.Forbes & Hemsl. (Rubiaceae) as a Chinese herbal plant. These cyclohexane-fused octahydroquinolizine alkaloids exhibited inhibitory effects on hepatitis C virus [27].

Renard-Nozaki et al. investigated the effects of some alkaloids in Amaryllidaceae, which were biosynthesized from *N*-benzylphenethylamine and their derivatives to be assessed against HSV-1 activity, as well as their antiviral mechanisms. The results showed that the synthesized apogalanthamine group possessed an anti-HSV-1 activity due to the related hexahydroindole ring with two functional hydroxyl groups. This hexahydroindole ring was responsible for antiviral activities and inhibition of HSV multiplication instead of extracellular viruses by reduction of viral DNA polymerase activity. In their study, lycorine and its derivative hippeastrine showed the highest antiviral activities among isolated alkaloids [28].

Amongst other traditional Chinese herbs, *Tetradium ruticarpum* (A. Juss.) T.G.Hartley (syn. *Evodia ruteacarpa*) (Rutaceae) was used to isolate three major quinazoline alkaloids named dehydroevodiamine, evodiamine, and rutaecarpine. The antiviral activities of these alkaloids were evaluated with showing inhibitory effects on H1N1-induced Regulated upon Activation, Normal T cell Expressed and Secreted (RANTES) production. Evodiamine exhibited the highest anti-H1N1 activity and inhibitory effect against H1N1 virus. This effect was also represented with rutaecarpine but not dehydroevodiamine. The results suggested the aforementioned alkaloids as potentially active compounds for the inhibition of H1N1-induced chemokine production as well as the suppression of chemotactic migration and chemokine-attracted leukocyte recruitment [29]. As another traditional Chinese herbal medicine with antiviral activities, *Tripterygium hypoglaucum* (H.Lév.) Hutch. (Celastraceae) showed promising antiviral effects. In a report by Ren et al., the anti-HSV-1 activity of root-extracted alkaloids was evaluated. These alkaloids decreased related UL30 and UL39 sequences and delayed early genes and US6 viral late gene transcription of HSV-1 genome, which led to the inhibition of HSV-1 activity [30].

Moradi et al. investigated the anti-influenza effects of extracted alkaloids from seeds of *Peganum harmala* L. (Nitrariaceae). The isolated alkaloids suppressed viral RNA replication and polymerase activity while not suppressing hemagglutination. Thus, the alkaloids of *P. harmala* were suggested as a potential agent against influenza A virus [31]. In a parallel study on the Chinese herbal plant *Sophora alopecuroides* L. (Fabaceae), the antiviral activity of five new quinolizidine-based alkaloids isolated from the seeds against the hepatitis B virus (HBV) was evaluated. These alkaloids were sparteineindolizine, matrine-indolizine, and epimeric normatrine-julolidine (with unusual skeletons). Anti-HBV activity of matrine-indolizine suggested them as potential antiviral agents [32]. Sun et al. isolated five indole alkaloids from *Isatis tinctoria* L. (syn. *Isatis indigotica*) (Brassicaceae), a traditional Chinese herb. All alkaloids exhibited anti-HSV-2 activities, with the structures of 3-hydroxy-3-acetonitrile-4-hydroxy-2-indolone, 1-methoxy-3-indoleacetonitrile, 3-indoleacetic acid, 3-indolealdehyde, and 1-methoxy-3-indolecarbaldehyde [33]. Besides, an indolizidine alkaloid from *L. radiata,* lycorine, at 15 nM concentration showed *in vitro* anti-SARS-CoV activities [34]. The lycorine along with other alkaloids, including tetrandrine, harmine, conessine, and emetine, showed antiviral activities against HCoV such as HCoV-OC43 and HCoV-NL63. In their study, lycorine was shown to inhibit the propagation and replication of HCoV-OC43 in the rat brain, probably through blocking viral proteases [35].

In another research, total alkaloids extracted from *Alstonia scholaris* (L.) R.Br. (Apocynaceae), another folk herbal plant that is used in China, exhibited anti-influenza effects. These alkaloids suppressed viral replication, increased the survival rate, and exhibited cytokine inhibitory effects at the mRNA/protein levels. They also deactivated the pattern recognition receptor and interferon-activated signal transduction pathway. Total alkaloids also ameliorated innate immune cell infiltration and improved lung histopathology, *in vivo* [36].

## 4. Alkaloids as Potential Phytochemicals against SARS-CoV-2

**Figure 1:**
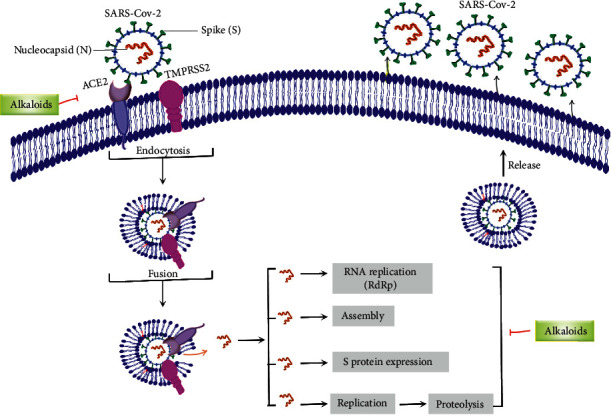
Major targets of alkaloids in combating SARS-CoV-2. ACE2: angiotensin-converting enzyme 2, TMPRSS2: transmembrane serine protease 2, and SARS-CoV-2: severe acute respiratory syndrome coronavirus-2.

Recent studies showed that alkaloids interact with the coronavirus structural proteins, including spike (S) glycoprotein and nucleocapsid (N) on the virus surface, as well as nonstructural angiotensin-converting enzyme 2 (ACE2) in the cell membrane, and, in turn, inhibit the enzymes involved in coronavirus replication, such as RdRp and 3-chymotrypsin-like protease (3CL^pro^) [37–39] (Figure 1). Wink suggested that highly DNA-interacting alkaloids, such as sanguinarine, chelerythrine, palmatine, chelidonine, berbamine, berberrubine, coptisine, dicentrine, jatrorrhizine, and berberine, could be suitable agents against SARS-CoV-2 [37]. Besides, a docking study on *Tinospora cordifolia* (Willd.) Hook.f. & Thomson (Menispermaceae) alkaloids, berberine (−7.3 Kcal/mol), and tetrahydropalmatine (−6.4 kcal/mol) showed a high binding affinity to 3CL^pro^ of SARS-CoV-2 [10]. Another docking study reported tryptanthrine (−8.2 kcal/mol), indirubin (−7.6 kcal/mol), indigo (−7.5 kcal/mol), and indican (−7.5 kcal/mol) as other alkaloids interacting with SARS-CoV-2 Mpro [40]. Besides, tryptanthrine and 5aR-ethyltryptanthrin isolated from methanolic extraction of *Strobilanthes cusia* (Acanthaceae) revealed anti-human coronavirus (HCoV) activities in a cytopathic effect reduction assay at 1.52 *μ*M and 2.60 *μ*M, respectively. In addition, tryptanthrine inhibited the HCoV-NL63 infectivity in human lung epithelial cells at 0.30 *μ*M with low cytotoxic effects on lung epithelial cells (CC50 = 173.2 *μ*M) [41]. On the other hand, due to the prominent antiviral and anti-inflammatory effects of indirubin and indigo, as two indole alkaloids, they can be considered as appropriate candidates for further COVID-19 treatment studies [42–44].

Also, Mpro was the main target for anti-SARS-CoV-2 effects of thalimonine, a pavine alkaloid, and sophaline D, a matrine-acetophenone alkaloid. According to the antitussive activities of thalimonine and antirhinovirus activities of sophaline D, these alkaloids are promising candidates for COVID-19 treatment [45].

In addition to the aforementioned alkaloids, three bisbenzylisoquinoline alkaloids of *Stephania tetrandra* S. Moore (Menispermaceae), including tetrandrine, cepharanthine, and fangchinoline, inhibited HCoV-OC43 24 hours after treatment at 5 *μ*M via reducing the expression of S and N proteins. The selectivity indices (CC50/IC50) of the tetrandrine, fangchinoline, and cepharanthine on MRC-5 human lung cells were 40.11, 11.46, and 13.63 *μ*M, respectively, which revealed their low levels of toxicity [46]. In addition to the aforementioned mechanism, cepharanthine showed anti-SARS-CoV-2 effects through the inhibition of RdRp, *in silico* [47]. Besides, cepharanthine improved the lung injuries, as a critical complication of COVID-19, with the effects on inflammatory signaling pathways. So, cepharanthine could be of promising candidates in combating COVID-19 [48]. Also, Maurya et al. showed that the S glycoprotein of SARS-CoV-2 and ACE2 are major targets of thebaine, berberine, and piperine. Despite the lower oral bioavailability of thebaine, its highest safety has been shown in comparison to other two alkaloids [39].

A docking study on the evaluation of interactions between alkaloids of African plants and 3CL^pro^ of coronaviruses showed that 10-hydroxyusambarensine, cryptoquindoline, cryptospirolepine, and chrysopentamine possessed stronger binding affinities than ritonavir and lopinavir [49]. Another study reported that *Cryptolepis sanguinolenta* alkaloids, including cryptomisrine, cryptospirolepine, and cryptoquindoline, showed acceptable interactions (>−8.5 kcal/Mol) with Mpro/RdRp of SARS-CoV-2. In their study, absorption, distribution, metabolism, and excretion (ADMET) property prediction showed that all *C. sanguinolenta* alkaloids have shown adequate gastrointestinal absorption and are able to cross the blood-brain barrier while possessing a low level of toxicity [50]. In addition, Zhang et al. showed two alkaloids, oxysophoridine, and lycorine with anti-SARS-CoV-2 effects in Vero-E6 cell culture at 0.31 and 0.18 *μ*M, respectively. In their study, inhibition of nucleotide biosynthesis was suggested for the aforementioned antiviral effects [51]. In addition to its anti-SARS-CoV-2 effects, lycorine also has prominent neuroprotective effects that can play a multifaceted role in the treatment of this disease due to the neurological complications of COVID-19 disease [52, 53]. Consequently, Bleasel and Peterson published a commentary on the potential of emetine and ipecac alkaloids as anti-SARS-CoV-2 agents [54]. Recently, the anti-HSV alkaloids, including emetine and homoharringtonine, have been introduced as potential agents in combating COVID-19. Their report was in line with that of Choy et al. in showing *in vitro* anti-SARS-CoV-2 activities of emetine and homoharringtonine, at 2.55 and 0.46 *μ*M, respectively [11, 55]. Additionally, Kumar et al. showed that emetine (2.3 ng/egg) inhibited SARS-CoV-2 replication via suppressing viral proteins such as viral polymerase in the infecting chicken embryonic model with IBV (chicken coronavirus) [56]. Also, another study showed that homoharringtonine strongly interacted with SARS-CoV-2 S protein among eleven anti-SARS-CoV alkaloids with a LibDock score of 109.11 kcal/mol [57].

Solanidine is a toxic steroid alkaloid of potato (Solanaceae) possessing a strong binding to SARS-CoV-2 Mpro and S proteins with a binding affinity of −7.0 and −9.1 kcal/mol, respectively [58]. The binding affinity of anisotine as a quinazoline alkaloid of the leaves of *Justicia adhatoda* L. (Acanthaceae) to SARS-CoV-2 Mpro was reported to be −7.9 kcal/mol that was stronger than that of darunavir and lopinavir, as positive control drugs [59]. Besides, Kar et al. showed the predicted the inhibitory constant (Ki) of anisotine interaction with Mpro, S proteins, and RdRp of SARS-CoV-2 was 1.90 *μ*M, 0.70 *μ*M, and 47.08 *μ*M, respectively [60].

In two *in silico* reports, Gurung et al. showed suitable binding affinity of the alkaloids such as 18-hydroxy-3-epi-alphayohimbine, alloyohimbine, asparagamine A, vincapusine, sophoridine, and lycorine to Mpro of coronaviruses, especially the SARS-CoV-2 [61, 62]. In another study, Gul et al. showed that the alkaloids such as dihydroergotamine and ergotamine possess acceptable inhibitory interactions with SARS-CoV-2 3CL^pro^ and RdRp [63]. Due to the anti-inflammatory effects, especially the neurological inflammation of ergotamine and dihydroergotamine, and the antiviral effects mentioned for them, these two ergot alkaloids could be introduced as promising candidates for the treatment of COVID-19 [64]. Also, Mustafa et al. showed that ergometrine and papaverine had the highest docking score in interaction with SARS-CoV-2 Mpro among alkaloids which has been approved to be safe by the FDA for use in various diseases [65].

In addition to their benefits to being used against COVID-19, the absorption, distribution, metabolism, excretion, and toxicity (ADMET) of alkaloids are limiting factors in their usage as anti-SARS-CoV-2 agents. Sanguinarine as a benzophenanthridine alkaloid, palmatine as an isoquinoline alkaloid, and tabersonine as a monoterpenoid indole alkaloid showed a good binding affinity to 3CL^pro^ and suitable ADMET properties. However, sanguinarine showed AMES toxicity [66]. Besides, sanguinarine, palmatine, and tabersonine have shown prominent inhibitory effects on inflammatory signaling pathways such as cyclooxygenase-2, mitogen-activated protein kinases, and NF-*κ*B, which have been shown to play a critical role in the development of SARS-CoV-2 complications. So, they could be introduced for further clinical trial studies on COVID-19 patients [67–69].

Besides, in another study, Joshi et al. showed that the (-)-asperlicin C had high binding affinity to 3CL^pro^ (−9.7 kcal/mol) and ACE2 (−9.5 kcal/mol), as well as oriciacridone F, with a high binding affinity to RdRp (−9.6 kcal/mol) [70]. In addition, tylophorine (0.018 *μ*M) as a phenanthroindolizidine alkaloid and 7-methoxycryptopleurine (<0.005 *μ*M) as a phenanthroquinolizidine alkaloid showed anti-SARS-CoV activities in Vero 76 cells via the inhibition of S and N proteins and 3CL^pro^ [71]. Transmembrane Protease Serine 2 (TMPRSS2) and cathepsin L are two host cell proteases that are required for binding the S protein of SARS-CoV-2 to host cells and entry. Vivek-Ananth et al. showed the alkaloids such as adlumidine and qingdainone with TMPRSS2 as well as oxoturkiyenine and 3*α*, 17*α*-cinchophylline and cathepsin L possess strong interaction and, thereby, are suitable agents against SARS-CoV-2 infection [72].

Envelope (E) protein is another structural protein of SARS-CoV-2 that plays critical roles in the assembly step of virus replication. Berbamine and its derivative (BE33) showed anti-SARS-CoV-2 activity through the inhibition of E protein at 14.50 and 0.94 *μ*M, respectively. These compounds decreased the lung injury induced by SARS-CoV-2E protein via reducing proinflammatory cytokines in the serum of C57BL/6 mice [73]. In addition to those structural proteins, nonstructural proteins (Nsps), especially Nsp15, have also played critical roles for SARS-CoV-2 replication. Some alkaloids such as ajmalicine (−8.1 kcal/mol), reserpine (−7.4 kcal/mol), berberine (−7.3 kcal/mol), and taspine (−7.3 kcal/mol) showed anti-SARS-CoV-2 activities via the inhibition of Nsp15 [74]. Berberine could be one of the promising candidates for the treatment of COVID-19 and reducing the side effects induced by SARS-CoV-2. In addition to its prominent antiviral effects on a variety of viruses, this isoquinoline alkaloid exhibited beneficial activities such as antioxidant, anti-inflammatory, immunomodulatory, neuroprotective, cardioprotective, nephroprotective, hepatoprotective, and anti-lung-injury effects [48, 75, 76].

Noscapine is a phthalideisoquinoline alkaloid from some species of the Papaveraceae family such as *Papaver somniferum* with antitussive and nonnarcotic effects. Several studies showed the noscapine and its derivatives, especially N-bromobenzyl derivative (23b), have strong and stable interaction with the Mpro enzyme of SARS-CoV-2 and, thereby, they are promising candidates for COVID-19 treatment [77, 78]. Fungi, especially genus *Aspergillus*, are one of the richest sources of alkaloids. Youssef et al. introduced fumigatoside E as a promising candidate for the treatment of COVID-19 because this pyrazinoquinazoline indole alkaloid, isolated from *Aspergillus fumigatus*, had a very strong interaction with ACE2 (−21.17 kcal/Mol). It also strongly interacted with microbial proteins such as DNA-gyrase, topoisomerase IV, and beta-lactamase [79]. Besides, norquinadoline A, as a fungal quinazoline alkaloid isolated from *Cladosporium* sp, showed strong interaction with ACE2 and papain-like protease (PLpro) with binding energies of −10.63 kcal/mol and −10.90 kcal/mol, respectively. This alkaloid had high safety and appropriate pharmacokinetic behavior in the predictive of ADMET properties [80, 81].

Marine natural products including polycyclic guanidine alkaloids, isolated from several species of marine sponges, show prominent biological and pharmacological effects. El-Demerdash et al. reported that crambescidin 786 and crambescidin 826 as two polycyclic guanidine alkaloids strongly interacted with SARS-CoV-2 proteins including Mpro, S proteins, N proteins, membrane glycoprotein, and Nsp 10 during a docking study. The highest binding energies of crambescidin 786 and crambescidin 826 were with Nsp 10 (−9.06 kcal/mol) and N protein (−8.01 kcal/mol), respectively. The results of these studies also showed that the presence of long-chain omega fatty acids in the structure of these alkaloids is critical for their strong interactions with SARS-CoV-2 proteins [82].

Caffeine, nicotine [83], and pseudojervine [84] via the interaction with ACE2 and amaranthin [85], speciophylline, cadambine [86], schizanthine Z, and schizanthine Y [87] via the interaction with Mpro showed anti-SARS-CoV-2 activities. In addition to the aforementioned agents involved in the pathogenesis of SARS-CoV-2, calcium ions also have shown vital roles for the entry of SARS-CoV-2 to the host cells. Berbamine also blocked the calcium transition, thereby showing anti-SARS-CoV-2 effects [88]. Also, inhibiting the calcium transition was one of the anti-SARS-CoV-2 mechanisms of four bisbenzylisoquinoline alkaloids including cepharanthine, hernandezine, neferine, and tetrandrine, which were selected from among 188 natural compounds studied for anti-SARS-CoV-2 activities. Besides, neferine at 10 *μ*M reduced the levels of relative viral RNA by 76.98% [89].

In a docking study, the alkaloids of *Argemone mexicana* L. (Papaveraceae) including protopine, allocryptopine, and 6-acetonyldihydrochelerythrine showed a promising interaction with RdRp [38]. Also, in another study on *Pilocarpus microphyllus* alkaloids (Rutaceae), epiisopiloturine showed a suitable interaction with Mpro (−7.0 kcal/mol). Nevertheless, this imidazolic alkaloid was detected as a mutagenic compound in the analysis of ADMET parameters (Ames test) [90].

Quinine, as a *Cinchona* alkaloid, showed antiviral activities against SARS-CoV-2 at EC90 of 38.8 *μ*M. Also, quinine had slight cytotoxic effects (CC50 > 100 *μ*M) on VERO E6 cell lines. In this line, Roza et al. showed that quinine interacted with Mpro (−6.2 kcal/mol) and S proteins (−5.7 kcal/mol). As fever is one of the most common side effects of COVID-19 and quinine has prominent antipyretic effects, this alkaloid could be introduced as a treatment for handling this complication of COVID-19 [91–93].

In addition, several clinical trials are ongoing on alkaloids such as colchicine (NCT04527562, NCT04392141, NCT04375202, NCT04355143, and NCT04360980), berberine (NCT04479202), and tetrandrine (NCT04308317). Therefore, based on the abovementioned studies, which showed their high efficacy in the treatment of COVID-19 disease, alkaloids could be introduced as hopeful anti-SARS-CoV-2 agents. The types of alkaloids, chemical structure, and anti-SARS-CoV-2 mechanisms obtained from *in vitro* and *in silico* studies are summarized in Tables 1 and 2, respectively.

## 5. Conclusions

The inherent complexity of SARS-CoV-2 makes it difficult for patients with COVID-19 to find effective treatments. The present study shows that alkaloids, as one of the most widespread natural compounds, hold out the hope for an effective treatment against COVID-19, due to their simultaneous effects on several therapeutic targets with prominent antiviral effects. Hence, marine/plant-derived alkaloids such as berberine, tetrandrine, cepharanthine, lycorine, ergotamine, crambescidin 786, palmatine, noscapine, and quinine with prominent anti-SARS-CoV-2 effects along with antipyretic, anti-inflammatory, antitussive and lung injury, immunomodulatory, and protective effects against neurotoxicity, cardiotoxicity, nephrotoxicity, and hepatotoxicity could be promising candidates for COVID-19 treatment [46, 48, 52, 64, 68, 76, 77, 79, 82, 93]. Therefore, according to the contents mentioned, extensive and comprehensive clinical studies on these compounds seem to be useful and necessary. Besides, the present study covers a much larger number of alkaloids with anti-SARS-CoV-2 effects and discusses the molecular mechanisms of these compounds in more detail [94, 95].

## Figures and Tables

**Table 1:** *In vitro* studies of alkaloids with potential anti-SARS-CoV-2 activity.

Alkaloid	Chemical structure	Mechanisms of action	Type of study	EC50	Ref.
7-Methoxycryptopleurine	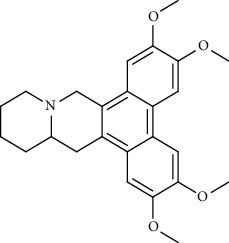	Blocking the S and N proteins, 3CL^pro^ inhibitor	*In vitro*	58 nM	[71]
Berbamine	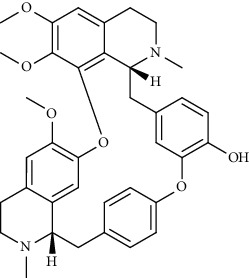	Blocking the E proteins and the calcium transition	*In vitro*	14.5 *μ*M, 2.3 mM	[73, 88]
Berbamine derivative (BE33)	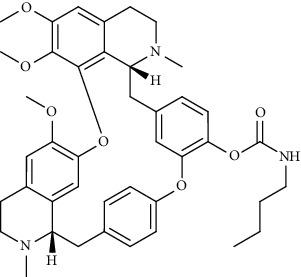	Blocking the E proteins	*In vitro*	0.94 *μ*M	[73]
Cepharanthine	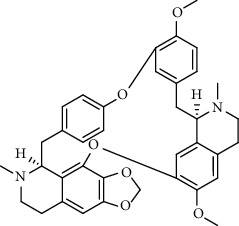	Blocking the expression of S and N proteins, RdRp inhibitor	*In vitro*	0.83 *μ*M	[46, 47]
Conessine	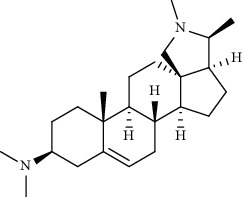	Mpro inhibitor	*In vitro*	2.34 *μ*M, 10.75 *μ*M	[35]
Emetine	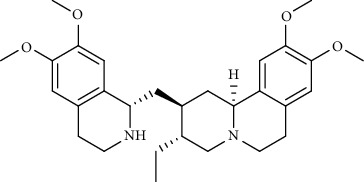	Mpro inhibitor	*In vitro*	2.55 *μ*M	[11, 55]
Fangchinoline	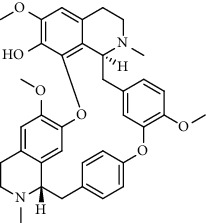	Blocking the expression of S and N proteins	*In vitro*	1.01 *μ*M	[46]
Harmine	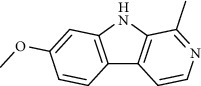	Mpro inhibitor	*In vitro*	1.9 *μ*M, 13.46 *μ*M	[35]
Hernandezine	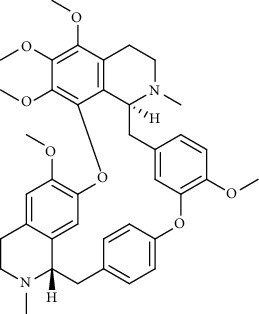	Blocking the calcium transition	*In vitro*	10 *μ*M	[89]
Homoharringtonine	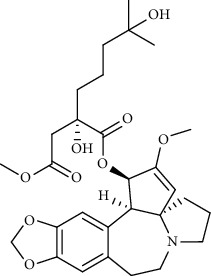	Blocking S proteins	*In vitro*	0.46 *μ*M	[11, 55, 57]
Lycorine	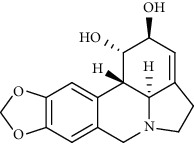	Mpro inhibitor	*In vitro*	15 nM, 0.15 *μ*M 0.47 *μ*M	[34, 35]
Neferine	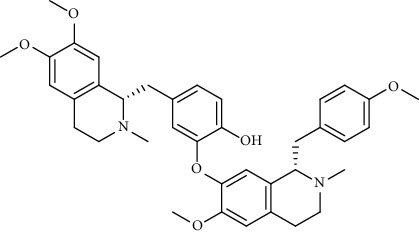	Decreasing the levels of relative viral RNA	*In vitro*	10 *μ*M	[89]
Oxysophoridine	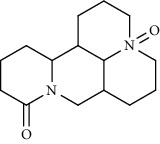	Nucleotide biosynthesis inhibitor	*In vitro*	0.31 *μ*M	[51]
Quinine	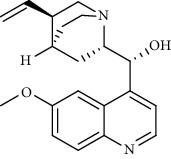	Mpro and S proteins inhibitor (*in silico* study)	*In vitro*	10.7 *μ*M	[91, 93]
Tetrandrine	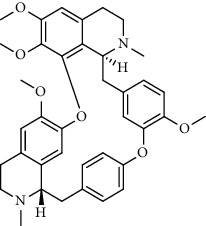	Blocking the expression of S and N proteins, Mpro inhibitor	*In vitro*	0.33 *μ*M, 0.29 *μ*M, 2.05 *μ*M	[35, 46]
Tylophorine	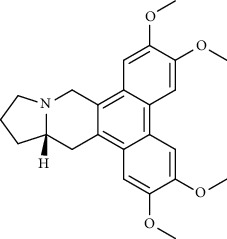	Blocking the S and N proteins, 3CL^pro^ inhibitor	*In vitro*	20 nM	[53]

3CL^pro^: 3-chymotrypsin-like protease, ACE2: angiotensin-converting enzyme 2, E proteins: envelope proteins, Mpro: main proteases, NI: not identified, N proteins: nucleocapsid proteins, Nsp15: nonstructural proteins, RdRp: RNA-dependent RNA polymerase, S proteins: spike proteins, TMPRSS2: transmembrane protease serine 2.

**Table 2:** *In silico* studies of alkaloids with potential anti-SARS-CoV-2 activity.

Alkaloid	Chemical structure	Mechanisms of action	Type of study	References
(-)-asperlicin C	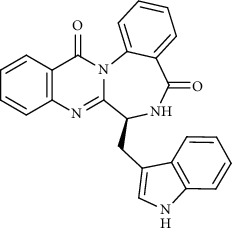	3CL^pro^ inhibitory, blocking ACE2	Molecular docking	[70]
3*α*,17*α*-Cinchophylline	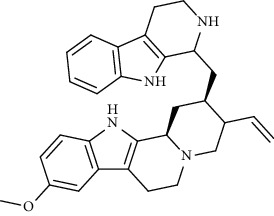	Cathepsin L inhibitor	Molecular docking	[72]
10-Hydroxyusambarensine	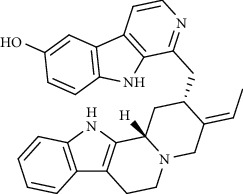	3CL^pro^ inhibitor	Molecular docking	[49]
18-Hydroxy-3-epi-alphayohimbine	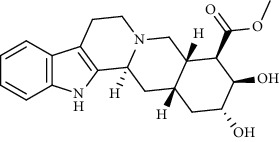	3CL^pro^ inhibitor	Molecular docking	[61]
6-Acetonyldihydrochelerythrine	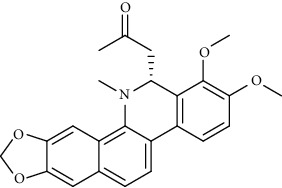	RdRp inhibitor	Molecular docking	[38]
Adlumidine	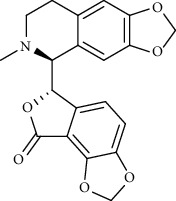	TMPRSS2 inhibitors	Molecular docking	[72]
Ajmalicine	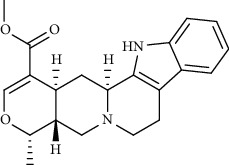	Blocking Nsp15	Molecular docking	[74]
Allocryptopine	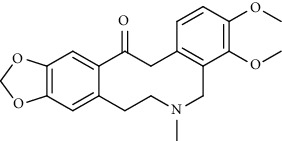	RdRp inhibitor	Molecular docking	[38]
Alloyohimbine	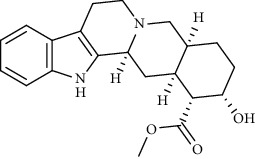	3CL^pro^ inhibitor	Molecular docking	[61]
Amaranthin	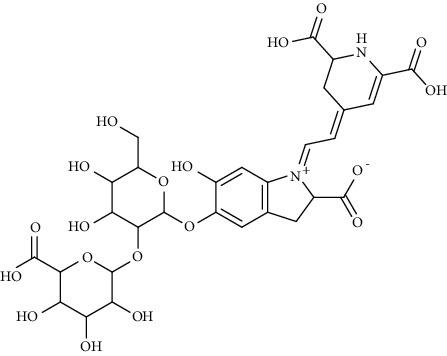	3CL^pro^ inhibitor	Molecular docking	[85]
Anisotine	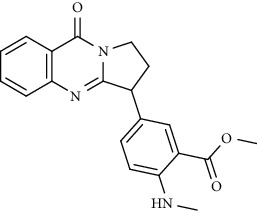	Mpro inhibitor	Molecular docking	[59]
Asparagamine A	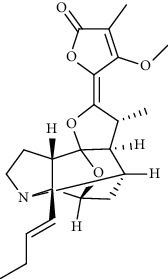	3CL^pro^ inhibitor	Molecular docking	[61]
Berberine	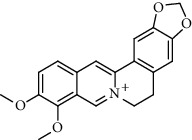	3CL^pro^ inhibitory, blocking ACE2 and Nsp15	Molecular docking	[10, 39, 74]
Cadambine	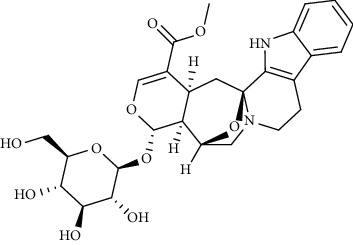	3CL^pro^ inhibitory	Molecular docking	[86]
Caffeine	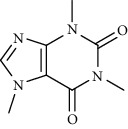	Blocking ACE2	Molecular docking	[83]
Chrysopentamine	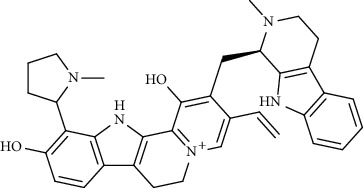	3CL^pro^ inhibitor	Molecular docking	[49]
Crambescidin 786	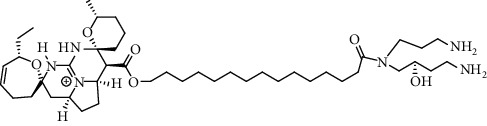	Blocking Nsp 10	Molecular docking	[82]
Crambescidin 826	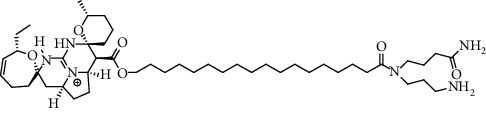	Blocking the N proteins	Molecular docking	[82]
Cryptomisrine	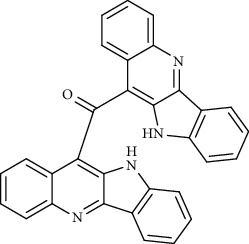	Mpro and RdRp inhibitor	Molecular docking	[50]
Cryptoquindoline	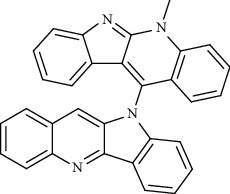	Mpro and RdRp inhibitor	Molecular docking	[49, 50]
Cryptospirolepine	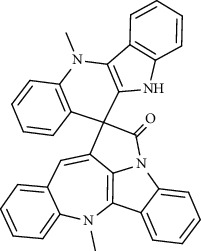	Mpro and RdRp inhibitor	Molecular docking	[49, 50]
Dihydroergotamine	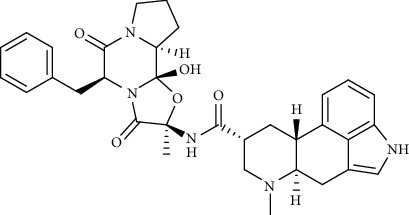	3CL^pro^ inhibitor	Molecular docking	[63]
Epiisopiloturine	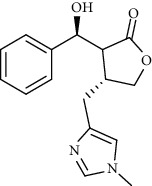	Mpro inhibitor	Molecular docking	[90]
Ergometrine	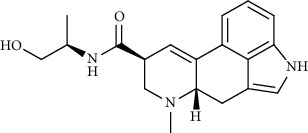	Mpro inhibitor	Molecular docking	[65]
Ergotamine	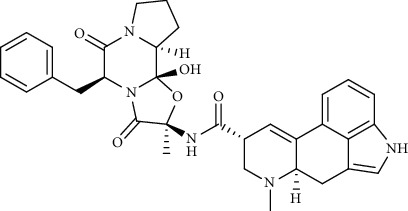	3CL^pro^ inhibitor	Molecular docking	[63]
Fumigatoside E	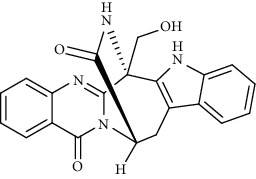	Blocking ACE2	Molecular docking	[79]
Indican	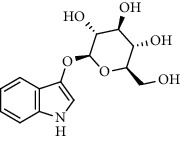	Mpro inhibitor	Molecular docking	[40]
Indigo	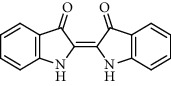	Mpro inhibitor	Molecular docking	[40]
Indirubin	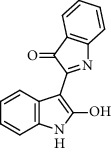	Mpro inhibitor	Molecular docking	[40]
Nicotine	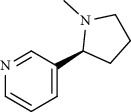	Blocking ACE2	Molecular docking	[83]
Norquinadoline A	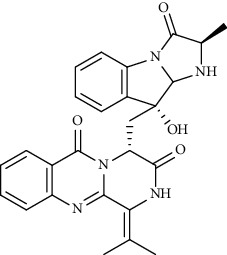	Blocking ACE2 and PLpro inhibitor	Molecular docking	[80, 81]
Noscapine	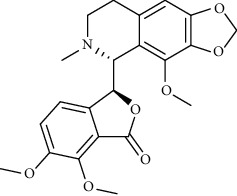	Mpro inhibitor	Molecular docking	[77]
Noscapine 23B	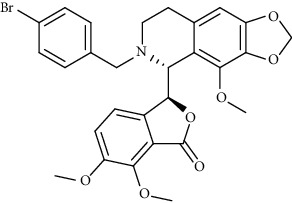	Mpro inhibitor	Molecular docking	[78]
Oriciacridone F	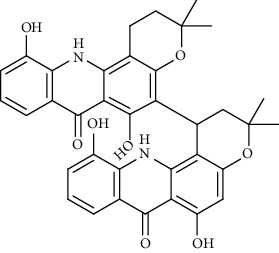	3CL^pro^ inhibitory, blocking ACE2	Molecular docking	[70]
Oxoturkiyenine	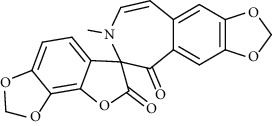	Cathepsin L inhibitor	Molecular docking	[72]
Palmatine	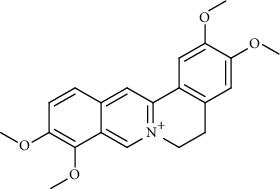	3CL^pro^ inhibitor	Molecular docking	[66]
Papaverine	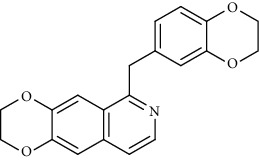	Mpro inhibitor	Molecular docking	[65]
Piperine	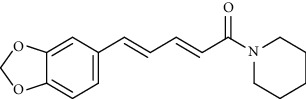	ACE2 blocker	Molecular docking	[39]
Protopine	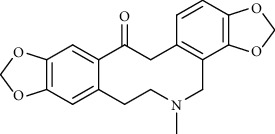	RdRp inhibitor	Molecular docking	[38]
Pseudojervine	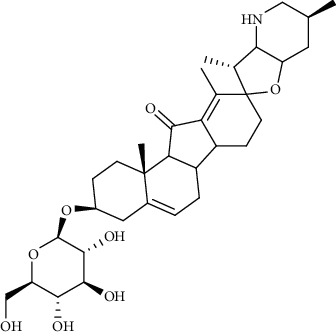	ACE2 blocker	Molecular docking	[84]
Qingdainone	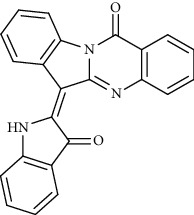	Inhibitors of TMPRSS2	Molecular docking	[72]
Reserpine	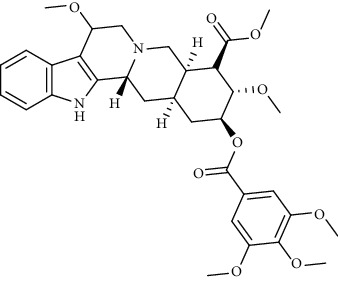	Blocking the Nsp15	Molecular docking	[74]
Sanguinarine	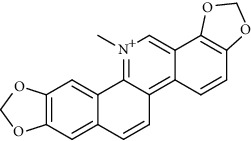	3CL^pro^ inhibitor	Molecular docking	[66]
Solanidine	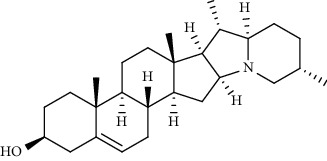	Blocking the S proteins, Mpro inhibitor	Molecular docking	[58]
Sophaline D	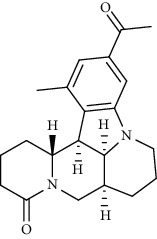	Mpro inhibitor	Molecular docking	[45]
Sophoridine	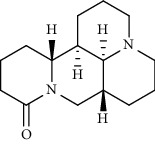	Mpro inhibitor	Molecular docking	[62]
Speciophylline	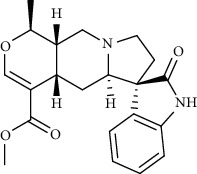	3CL^pro^ inhibitor	Molecular docking	[86]
Tabersonine	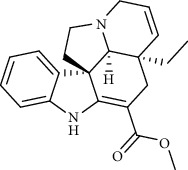	3CL^pro^ inhibitor	Molecular docking	[66]
Taspine	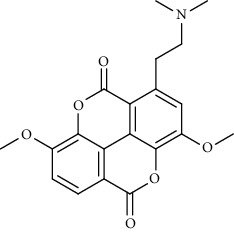	Blocking Nsp15	Molecular docking	[74]
Tetrahydropalmatine	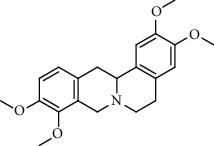	3CL^pro^ inhibitor	Molecular docking	[10]
Thalimonine	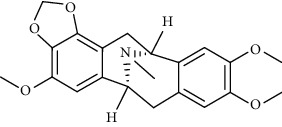	Mpro inhibitor	Molecular docking	[45]
Thebaine	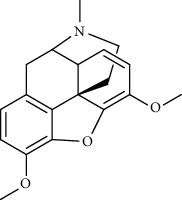	ACE2 blocker	Molecular docking	[39]
Tryptanthrine	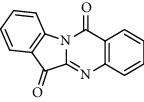	Mpro inhibitor	Molecular docking	[40]
Vincapusine	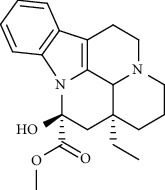	3CL^pro^ inhibitor	Molecular docking	[61]

3CL^pro^: 3-chymotrypsin-like protease, ACE2: angiotensin-converting enzyme 2, E proteins: envelope proteins, Mpro: main proteases, NI: not identified, N proteins: nucleocapsid proteins, Nsp15: nonstructural proteins, RdRp: RNA-dependent RNA polymerase, S proteins: spike proteins, TMPRSS2: transmembrane protease serine 2.

## Data Availability

No data were used to support this study.
